# Development of a Quantitative Loop-Mediated Isothermal Amplification Assay for the Rapid Detection of Novel Goose Parvovirus

**DOI:** 10.3389/fmicb.2017.02472

**Published:** 2017-12-12

**Authors:** Jing Yang, Hao Chen, Zhenzhong Wang, Xianglong Yu, Xiaoyu Niu, Yi Tang, Youxiang Diao

**Affiliations:** ^1^College of Animal Science and Technology, Shandong Agricultural University, Tai’an, China; ^2^Shandong Provincial Key Laboratory of Animal Biotechnology and Disease Control and Prevention, Tai’an, China; ^3^Shandong Provincial Engineering Technology Research Center of Animal Disease Control and Prevention, Tai’an, China

**Keywords:** novel goose parvovirus, EvaGreen^®^ dye, quantitative loop-mediated isothermal amplification (qLAMP), polymerase chain reaction (PCR), VP3 gene

## Abstract

An infectious disease characterized with short bills and protruding tongues has attacked to meat ducks in China since March 2015, which has caused ducks poor growth and enormous economic losses to duck industry of China. It was eventually proved to be caused by parvovirus after pathogen isolation and identification. As the genomic sequence analysis showed, this pathogen shared 90.8–94.6% of nucleotide identity with goose parvovirus (GPV), and it was called duck-origin novel goose parvovirus (N-GPV). In this study, a quantitative loop-mediated isothermal amplification (qLAMP) assay was developed for the rapid diagnosis of N-GPV. A set of four specific primers, two inner and two outer, were designed targeting at VP3 gene, which could be completed within 60 min at 65°C in water bath or on a real-time PCR instrument for quantitative analysis. Specificity test of LAMP assay showed that there was no cross-reactivity between N-GPV and other duck pathogens, and the detection limit of qLAMP assay was 1.0 × 10^2^ copies/μL. The repeatability of this method was confirmed by inter-assay and intra-assay tests with variability ranging from 0.74 to 2.25%. The results have indicated that the qLAMP assay was a simple, rapid, accurate, sensitive, and specific method for detecting N-GPV, especially on field detection.

## Introduction

Waterfowl parvovirus causes high morbidity and mortality in geese and Muscovy ducks, and mortality rate ranges from 10 to 80%. These diseases can lead to enteric symptoms, watery diarrhea, prostration, and growth retardation, thus resulting in serious economic losses to waterfowl industry (Glávits et al., 2005). In 2015, a disease, which emerged in France and Poland in the early 1970s in mule and Muscovy ducks was first reported in Chinese mainland (Chen et al., 2015). It mainly caused short beak, protruding tongues, fragile tibia and pteroid, and growth retardation to commercial ducks. The morbidity rate ranges from 10 to 30%, and even up to 50%, thereby resulting in enormous economic losses to duck industry of China. In order to identify the exact pathogen that caused this disease, pathogen isolation and identification and genomic sequence analysis were carried out. Isolated pathogen was detected by polymerase chain reaction (PCR) assay, and the result showed that only GPV was positive; Genomic sequence analysis showed that this new pathogen shared 90.8–94.6% of nucleotide identity with goose parvovirus (GPV). These two studies above indicated that this new pathogen shared higher homology with other GPV strains. Therefore, it was called duck-origin novel goose parvovirus (N-GPV) (Chen et al., 2016). In common with other waterfowl parvovirus, N-GPV was also a member of the *Parvoviridae* family with a single-stranded DNA genome, and has a small, nonenveloped, icosahedral capsid packaging a single-stranded DNA genome of approximately 5,100 nucleotides. There are two open reading frames (ORFs) encoding non-structural proteins (NS), and capsid proteins (VP) (Chen et al., 2015).

Recently, with advances in virological diagnositic techniques, a novel nucleic acid amplification method—loop-mediated isothermal amplification (LAMP) has attracted intense attention, which relies on auto-cycling strand displacement DNA synthesis by the *Bst* DNA polymerase large fragment with high strand displacement activity and a set of specific primers that recognizes six distinct sequences in the target DNA (Notomi et al., 2000). In addition, LAMP has been considered as a time-saving, low-cost, highly specific and sensitive method (Chotiwan et al., 2017), which can be completed within 60 min under condition of constant temperature, and it has been established to detect GPV, Muscovy duck parvovirus (MDPV), porcine parvovirus (PPV), canine parvovirus (CPV), and others targeting at VP gene (Cho et al., 2006; Chen et al., 2009; Ji et al., 2010; JinLong et al., 2010). Results of LAMP can be judged by either turbidity, or end-products that can be visible to naked eyes with fluorescent reagents such as SYBR Green I (Tomita et al., 2008).

Lately, a novel Eva Green-based quantitative LAMP assay has become popular (Wang et al., 2006; Lee et al., 2017). Compared with SYBR Green I-based quantitative LAMP assay, Eva Green dye^®^ is less inhibitory to PCR and less likely to cause nonspecific amplification (Ihrig et al., 2006). Meanwhile, aerosol pollution of this method can be avoided due to tubes closed. After amplification, results can be analyzed by amplification curve, turbidity, or fluorescence of end-products under ultraviolet light, thus judging whether target DNA is amplified (Mori et al., 2004).

In this study, a detection method quantitative loop-mediated isothermal amplification (qLAMP) for amplifying VP3 gene of N-GPV was established. Amplification curves and good linear relationship have been obtained. Specificity of this method was determined by GPV and other duck-origin viruses, such as duck plague virus, duck tembusu virus, duck hepatitis virus, duck reovirus, Muscovy duck parvovirus, and H9N2-AIV. In addition, sensitivity of qLAMP was carried out with plasmid substances after ten-fold series dilutions. These test results above demonstrated that this method has advantages with strong specificity, high sensitivity, easy operated, and low cost so that is valuable and suitable for clinical application in basic level production.

## Materials and Methods

### Viruses and Samples

N-GPV used in this study was isolated from morbid ducks liver in Liaocheng city, Shandong province, China (named SDLC strain). GPV (HN strain), duck plague virus (ZB strain), duck tembusu virus (GL strain), duck hepatitis virus (LC strain), duck reovirus (HB strain), Muscovy duck parvovirus (GX strain), and duck-origin H9N2-AIV (GT strain) were preserved by the Research Institute of Poultry Disease of Shandong Agricultural University. Sixty clinical liver samples and 60 cloacal swabs were collected from dead ducks and clinical healthy ducks respectively in Shandong, Jiangsu, and Henan province of China in different large-scale duck farms.

### Virus Isolation

The liver samples were homogenized in dulbecco’s modified eagle medium (DMEM), frozen and thawed three times, then centrifuged at 5000 ×*g* for 15 min. The supernatant were filtered through a 0.22 μm filter, and the filtrate was inoculated into duck embryo fibroblasts (DEF) cells. The culture supernatant was harvested after 5 days post-inoculated for three passages, and detected by PCR.

### Extraction of DNA and RNA

DNA was extracted by TIANamp Genomic DNA Kit (TIANGEN biotech, Beijing, China). RNA was extracted by MiniBEST Universal RNA Extraction Kit (TaKaRa, Dalian, China).

### Sequence Analysis

**FIGURE 1:**
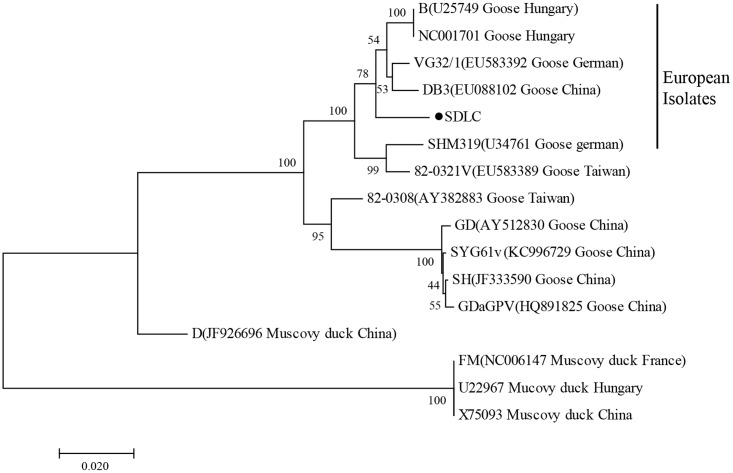
Phylogenetic relationship between SDLC (●) and other waterfowl parvovirus strains based on the VP3 gene in the phylogenetic tree built using the neighbor-joining method.

VP3 gene of N-GPV, GPV, and MDPV were aligned by the MegAlign software, and phylogenetic tree was constructed with MEGA version 7.0 using the neighbor-joining method (**Figure 1**). The reference waterfowl parvovirus isolates are listed in **Table 1**.

**Table 1:** Description of waterfowl parvovirus isolates involved in this study.

Isolates	Accession number	Host	Separatum
B	U25749	Goose	Hungary
NC001701	NC001701	Goose	Hungary
VG32/1	EU583392	Goose	German
DB3	EU088102	Goose	China
SDLC01	KT343253	Duck	China
SHM319	U34761	Goose	German
82-0321V	EU583389	Goose	Taiwan
82-0308	AY382883	Goose	Taiwan
GD	AY512830	Goose	China
SYG61V	KC996729	Goose	China
SH	JF333590	Goose	China
GDAGPV	HQ891825	Goose	China
D	JF926696	Muscovy duck	China
FM	NC006147	Muscovy duck	France
U22967	U22967	Muscovy duck	Hungary
X75093	U22967	Muscovy duck	China

### Primer Design

A set of four primers and PCR primers were designed based on the VP3 gene of N-GPV using an online program (PrimerExplorer V4, http://primerexplorer.jp/elamp4.0.0/index.html) and Primer 5.0 software respectively. Primers including two inner (FIP and BIP), two outer (F3 and B3), and PCR primers are shown in **Table 2**.

**Table 2:** Primers for quantitative loop-mediated isothermal amplification (qLAMP) assay and polymerase chain reaction (PCR) assay.

Primers		Squences (5′–3′)	Position
QLAMP	F3	ACCGAAGACTGATGGCAAAT	4266–4285
	B3	TCCCACACCATCTCTACTGT	4438–4457
	FIP	GATGAACACCTGCGGTGGTGG-CCATCCTTCTCCGAATCTCG	–
	BIP	ATACACCAGTGCCTGCAGACC-GGCCCGTAGAGTACTGAGT	–
PCR	F	GAGCATCAACTCCCGTATGTCC	3466–3487
	R	CTACTTCCTGCTCGTCCGTGA	4106–4126

### Preparation of Standard Substance

Outer primers were used to amplify target DNA, and the amplified PCR products were purified with a gel extraction kit (Takara, Dalian, China), cloned into the pMD18-T vector (Takara, Dalian, China) and transformed into *E. coli* DH5α competent cells. Clones were sequenced by BGI Tech (BGI Tech, Shenzhen, China), and analyzed results. High fidelity clone was cultured and extracted plasmid with Pureplasmid Mini Kit (CWBIO, Beijing, China). Meanwhile, 1.0 × 10^7^ copies/μL–1.0 × 10^2^ copies/μL substances were used for constructing standard curve.

### QLAMP Conditions

The qLAMP assay was conducted in a 25 μL reaction system, which containing 1 μL FIP and BIP primers (80 μM of each), 1 μL F3 and B3 primers (10 μM of each), 2.5 μL 10 × Isothemal amplification buffer, 1.5 μL 100 mM MgSO_4_, 3.5 μL 10 mM dNTP Mix, 1.25 μL 20 × EvaGreen^®^ dye, 1 μL Bst DNA polymerase, 1 μL target DNA, 11.5 μL nuclease-free water, and performed using 7300 Real time PCR System (Applied Biosystems, Foster City, CA, United States). Mixtures were incubated at 65°C in water bath for 60 min, and terminated by heating at 80°C for 20 min (followed by NEB typical LAMP protocol), or carried out on 7300 Real time PCR system followed by procedure: 60 cycles of 64°C 10 s and 65°C 50 s, fluorescence signals were collected at the end of 65°C step, and the cutoff point for Tt (time threshold, a cycle indicates 1 min) value was determined as 50 by the previous reported method(Tang et al., 2016). Data was analyzed by SDS software program (version 1.4). The qLAMP results were also analyzed by gel electrophoresis with 2% agarose gel, and observed under UV light.

### PCR Conditions

The PCR assay was conducted in 20 μL reaction system, containing 10 μL 2 × Ex Taq Mix, 6 μL ddH_2_O, 1 μL forward primer, 1 μL forward primer, 1 μL reverse primer, and 2 μL DNA, and performed using PCR System (Applied Biosystems, Foster City, CA, United States). PCR conditions were as follows: 95°C for 5 min, 30 cycles of denaturation (95°C for 30 s), annealing (55°C for 30 s), and extension (72°C for 30 s), followed by a final extension at 72°C for 10 min.

### Sensitivity of qLAMP Assay

Ten-fold series dilutions (10^7^–10^0^ copies/μL) of standard substance were used as templates for qLAMP amplification to confirm its sensitivity.

### Specificity of qLAMP Assay

Quantitative loop-mediated isothermal amplification assay was carried out using different viruses including GPV, duck plague virus, duck tembusu virus, duck hepatitis virus, duck reovirus, Muscovy duck parvovirus, and H9N2-AIV to validate specificity of this method.

### Comparation of PCR Assay, qLAMP Assay, and Virus Isolation

Sensitivity test was performed to compare PCR and LAMP assay, and plasmid with different dilutions was used as templates for PCR detection. F3, B3 Primers for PCR amplification were shown in **Table 2**. PCR results were electrophoresed in 2% TAE agarose gel and observed in the UV transilluminator.

### Reproducibility of qLAMP Assay

To confirm that the qLAMP detection method has good reproducibility to detect N-GPV, tenfold series dilutions of plasmid substances, from 1.0 × 10^7^ copies/μL to 1.0 × 10^0^ copies/μL were used. For the intra-assay test, triplicate from each dilution were tested in the same run. For the inter-assay test, samples from each dilution were tested in three independent runs. The results were evaluated by coefficient of variation.

### Clinical Samples Detection

Sixty clinical liver samples and 60 cloacal swabs from dead ducks and clinical healthy ducks respectively were collected to test reliability of qLAMP, PCR assay, and virus isolation assay.

## Results

### Phylogenetic Analysis of VP3 Genes

To determine genetic relationship between SDLC and other GPV and MDPV strains, phylogenetic tree of VP3 genes was constructed. Phylogenetic trees based on VP3 genes showed that the SDLC strains were in the same branch with European GPV and vaccine isolates, and shared 93.4–98.9% identity with GPV isolates, while only shared 80.4–88.7% with MDPV, which indicated that evolutionary relationship of SDLC strain is closer to GPV stains, and N-GPV is a novel variant of GPV.

### Preparation of Standard Substance and Establishment of Standard Curve

**FIGURE 2:**
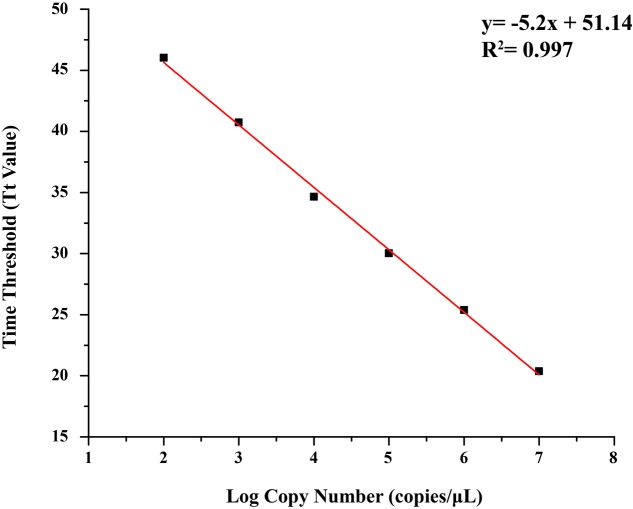
Standard curve of quantitative loop-mediated isothermal amplification (qLAMP) assay using tenfold series dilutions of plasmid substances in TE buffer (1.0 × 10^7^ copies/μL–1.0 × 10^2^ copies/μL).

Concentration of positive plasmid was 253.50 ng/μL, determined by DS-11 Spectrophotometer (Denovix, America), and the copy number was 7.91 × 10^10^ copies/μL. The standard curve was generated by Tt values obtained from this study, and a good linear relationship was established between the log of the plasmid copy numbers (copies/μL) and the Tt values (*R*^2^= 0.997), with a regression line revealing an average intercept of 51.14 and an average slope of –5.2 (**Figure 2**).

### Sensitivity of qLAMP Assay

**FIGURE 3:**
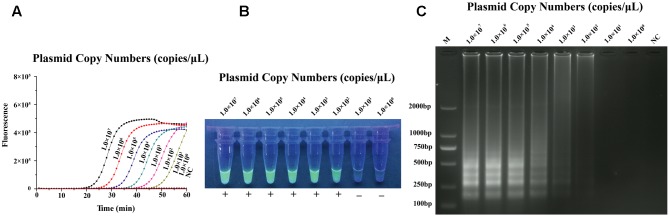
Sensitivity test results of qLAMP assay using tenfold series dilutions of plasmid substances in TE buffer (1.0 × 10^7^ copies/μL–1.0 × 10^0^ copies/μL) **(A)** Amplification plots of different dilutions plasmid substances. **(B)** Fluorescence of end-products in the UV light with EvaGreen^®^ dye. **(C)** Results of Agarose gel electrophoresis.

Performed by ten-fold series dilutions (1.0 × 10^7^–1.0 × 10^0^ copies/μL) of standard substance, the qLAMP method for N-GPV detection has the lowest limit of 10^2^ copies, and no amplification signals were observed in negative control (NC) (**Figure 3A**). In our strategy, the results of qLAMP assay could not only be evaluated by naked eyes at the endpoint with EvaGreen^®^ fluorescent dye in the UV light (**Figure 3B**) and agarose gel electrophoresis (**Figure 3C**), it could also be quantitatively monitored using the real-time PCR system during the reaction.

### Specificity of qLAMP Assay

**FIGURE 4:**
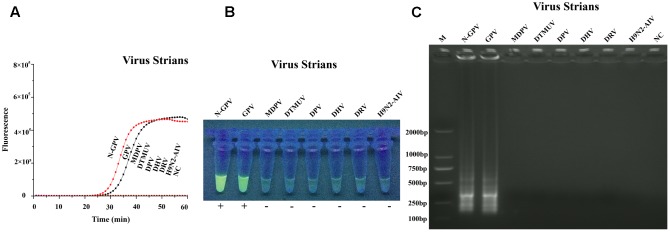
Specificity test results of qLAMP assay using different virus strains. **(A)** Amplification plots of different virus strains. **(B)** Fluorescence of end-products in the UV light with EvaGreen^®^ dye. **(C)** Results of Agarose gel electrophoresis.

Specificity test of qLAMP method was carried out by seven duck-origin viruses and a goose-origin virus (mentioned in virus and samples). The results showed that our assay could only detect N-GPV and GPV, while no amplification signals were detected in other viruses (**Figure 4A**). The specificity of qLAMP assay could also be confirmed by green fluorescence in the UV light (**Figure 4B**), and typical ladder pattern seen in the agarose gel electrophoresis (**Figure 4C**). In addition, no positive results were obtained in negative control (NC), which indicates that this qLAMP assay has a good specificity for N-GPV detection.

### Reproducibility of qLAMP Assay

In the intra-assay test, the coefficient of variation of the Tt values varied from 0.74 to 1.81%; In the inter-assay test, the coefficient of variation of the Tt values varied from 0.82 to 2.25% (**Table 3**). These results revealed that the qLAMP method has a high reproducibility and excellent stability in detecting N-GPV.

**Table 3:** Reproducibility analysis of qLAMP assay.

Copy numbers (copies/μL)	Intra-assay variability of Tt value			Inter-assay variability of Tt value		
	Mean	SD	CV (%)	Mean	SD	CV (%)
1.0 × 10^7^	20.37	0.15	0.74	20.36	0.17	0.82
1.0 × 10^6^	25.38	0.23	0.91	25.16	0.49	1.96
1.0 × 10^5^	30.03	0.54	1.81	30.00	0.50	1.96
1.0 × 10^4^	34.65	0.52	1.50	34.49	0.78	2.25
1.0 × 10^3^	41.11	0.56	1.37	40.72	0.38	2.05
1.0 × 10^2^	46.02	0.73	1.58	46.33	0.57	1.22

*CV, Coefficient Variation; SD, Standard Deviation (*n* = 3).*

### Contrast Experiment of qLAMP and PCR Assay

Different dilution plasmids (1.0 × 10^7^–1.0 × 10^0^ copies/μL) were used as templates for LAMP and PCR amplification. Results showed that the detection limit of qLAMP assay was 10^2^ copies, while PCR assay was 10^4^ copies, which indicated the sensitivity of LAMP method was 100 times higher than PCR method.

### Clinical Samples Detection

Loop-mediated isothermal amplification, PCR, and virus isolation assay were performed on clinical samples and cloacal swabs of ducks from different regions of China. The results were shown by statistical analysis in **Table 4**. The results showed that the consistency of these three methods in every sample. The sensitivity of qLAMP assay was the highest, and there was no positive amplification in negative control in qLAMP assay, PCR, and virus isolation assay.

**Table 4:** Statistical results of PCR, qLAMP, and virus isolation assay.

Origin				PCR				
				+		–		
			Virus isolation	+	–	+	–	Total
Livers		+		3	25	0	23	51
	QLAMP	–		0	0	0	9	9
			Total	3	25	0	32	60
			
			**Virus isolation**	**+**	**–**	**+**	**–**	**Total**
			
Cloacal		+		4	33	0	17	54
swabs	QLAMP	–		0	0	0	6	6
			Total	4	33	0	23	60

## Discussion

Currently, as a laboratory assay and conventional method, PCR is used most widely. However, not only does it require expensive equipments and much time, but also it has a high requirement for experiment conditions. Meanwhile, although the Taqman-based real-time PCR assay has been established for the detection of N-GPV, it is time-consuming and costly; In addition, the detection limit (8.8 × 10^1^ copies/μL) is almost the same as qLAMP assay (1.0 × 10^2^ copies/μL) (Niu et al., 2016), so the rapid, simple, reliable, and cost-efficient qLAMP assay is definitely a good choice in detecting N-GPV.

Although traditional SYBR Green I-based LAMP assay is a good method with high sensitivity and specificity, adding fluorescence dye after reaction would cause aerosol pollution, thus resulting in a false positive. However, SYBR Green I would inhibit PCR amplification if added before LAMP reaction (Eischeid, 2011). So, the novel EvaGreen-based quantitative LAMP assay has overcome these disadvantages and is more suitable for further application. This method could also be carried out in a water bath or on a real-time PCR instrument for quantitative analysis, and results can be easily observed by turbidity, fluorescence in the UV by naked eyes and agarose gel electrophoresis.

This study was the first to report the EvaGreen-based LAMP assay for detecting N-GPV. Based on the data above, the detection limit of this method was as low as 1.0 × 10^2^ copies/μL, especially the intra and inter-assay variations, — just 0.74–2.25% only. In addition, the specificity of this method was 100 times higher than traditional PCR assay, and was almost the same as qPCR assay.

Novel goose parvovirus is newly discovered in recent years, which mainly causes ducks growth retardation and high infection rate to meat ducks, while GPV mainly causes serious death to goslings. According to the view of homology, the N-GPV strain is closer to European GPV and vaccine isolates, while separated from Asian isolates. Therefore, it is clear that N-GPV is a member of GPV-related parvovirus. N-GPV and GPV are almost indistinguishable using this method due to higher nucleotide homology, but it’s also a great method for the detection of N-GPV, because these two pathogens are from different poultry.

## Conclusion

The qLAMP assay has proved to be a highly sensitive, specific, and reliable diagnostic method that can detect N-GPV in a simple, rapid, and cost-efficient way. It could be undoubtedly used as a point-of-care strategy for clinical laboratories to prevent diseases from breaking out (Notomi et al., 2015).

## Author Contributions

Conceived and designed the experiments: YD, YT, and JY. Performed the experiments: JY, HC, and ZW. Analyzed the data: JY, XY, and XN. Contributed reagents/materials/analysis tools: JY and HC. Wrote the paper: JY.

## Conflict of Interest Statement

The authors declare that the research was conducted in the absence of any commercial or financial relationships that could be construed as a potential conflict of interest.

## References

[B1] Chen H. Dou Y. Tang Y. Zhang Z. Zheng X. Niu X. (2015). Isolation and genomic characterization of a duck-origin GPV-related parvovirus from cherry valley ducklings in China. *PLOS ONE* 10:e0140284. 10.1371/journal.pone.0140284 26465143 PMC4605506

[B2] Chen H. Tang Y. Dou Y. Zheng X. Diao Y. (2016). Evidence for vertical transmission of novel duck-origin goose parvovirus-related parvovirus. *Transbound. Emerg. Dis.* 63 243–247. 10.1111/tbed.12487 26890433

[B3] Chen H. T. Zhang J. Yang S. H. Ma L. N. Ma Y. P. Liu X. T. (2009). Rapid detection of porcine parvovirus DNA by sensitive loop-mediated isothermal amplification. *J. Virol. Methods* 158 100–103. 10.1016/j.jviromet.2009.02.005 19428576 PMC7112806

[B4] Cho H.-S. Kang J.-I. Park N.-Y. (2006). Detection of canine parvovirus in fecal samples using loop-mediated isothermal amplification. *J. Vet. Diagn. Invest.* 18 81–84. 16566261 10.1177/104063870601800111

[B5] Chotiwan N. Brewster C. D. Magalhaes T. Weger-Lucarelli J. Duggal N. K. Rückert C. (2017). Rapid and specific detection of Asian- and African-lineage Zika viruses. *Sci. Transl. Med.* 9 1–14. 10.1126/scitranslmed.aag0538 28469032 PMC5654541

[B6] Eischeid A. C. (2011). SYTO dyes and evagreen outperform SYBR green in real-time PCR. *BMC Res. Notes* 4:263. 10.1186/1756-0500-4-263 21798028 PMC3162529

[B7] Glávits R. Zolnai A. Szabó E. Ivanics E. Zarka P. Palya T. M. V. (2005). Comparative pathological studies on domestic geese (anser anser domestica) and muscovy ducks (cairina moschata) experimentally infected with parvovirus strains of goose and muscovy duck origin. *Acta Vet. Hung.* 53 73–89. 10.1556/AVet.53.2005.1.8 15782661

[B8] Ihrig J. Lill R. Mühlenhoff U. (2006). Application of the DNA-specific dye evagreen for the routine quantification of DNA in microplates. *Anal. Biochem.* 359 265–267. 10.1016/j.ab.2006.07.043 16962553

[B9] Ji J. Xie Q. M. Chen C. Y. Bai S. W. Zou L. S. Zuo K. J. (2010). Molecular detection of muscovy duck parvovirus by loop-mediated isothermal amplification assay. *Poult. Sci.* 89 477–483. 10.3382/ps.2009-00527 20181863

[B10] JinLong Y. Rui Y. AnChun C. MingShu W. LiZhi F. SongQuan Y. (2010). A simple and rapid method for detection of *Goose Parvovirus* in the field by loop-mediated isothermal amplification. *Virol. J.* 7:14. 10.1186/1743-422X-7-14 20092637 PMC2829533

[B11] Lee S. H. Baek Y. H. Kim Y. H. Choi Y. K. Song M. S. Ahn J. Y. (2017). One-pot reverse transcriptional loop-mediated isothermal amplification (RT-LAMP) for detecting MERS-CoV. *Front. Microbiol.* 7:2166. 10.3389/fmicb.2016.02166 28119682 PMC5220095

[B12] Mori Y. Kitao M. Tomita N. Notomi T. (2004). Real-time turbidimetry of LAMP reaction for quantifying template DNA. *J. Biochem. Biophys. Methods* 59 145–157. 10.1016/j.jbbm.2003.12.005 15163526

[B13] Niu X. Chen H. Yang J. Yu X. Ti J. Wang A. (2016). Development of a TaqMan-based real-time PCR assay for the detection of Novel GPV. *J. Virol. Methods* 237 32–37. 10.1016/j.jviromet.2016.08.006 27523295

[B14] Notomi T. Mori Y. Tomita N. Kanda H. (2015). Loop-mediated isothermal amplification (LAMP): principle, features, and future prospects. *J. Microbiol.* 53 1–5. 10.1007/s12275-015-4656-9 25557475

[B15] Notomi T. Okayama H. Masubuchi H. Yonekawa T. Watanabe K. Amino N. (2000). Loop-mediated isothermal amplification of DNA. *Nucleic Acids Res.* 28:E63 10.1093/nar/28.12.e63 PMC102748 10871386

[B16] Tang Y. Yu X. Chen H. Diao Y. (2016). An immunoassay-based reverse-transcription loop-mediated isothermal amplification assay for the rapid detection of avian influenza H5N1 virus viremia. *Biosens. Bioelectron.* 86 255–261. 10.1016/j.bios.2016.06.063 27376196

[B17] Tomita N. Mori Y. Kanda H. Notomi T. (2008). Loop-mediated isothermal amplification (LAMP) of gene sequences and simple visual detection of products. *Nat. Protoc.* 3 877–882. 10.1038/nprot.2008.57 18451795

[B18] Wang W. Chen K. Xu C. (2006). DNA quantification using evagreen and a real-time PCR instrument. *Anal. Biochem.* 356 303–305. 10.1016/j.ab.2006.05.027 16797474

